# Utility of PCR in Patients with *Strongyloides stercoralis* and HTLV-1 Coinfection in French Guiana

**DOI:** 10.4269/ajtmh.19-0082

**Published:** 2019-08-19

**Authors:** Romain Blaizot, Stephane Simon, Jean Brottier, Denis Blanchet, Paul Brousse, Rachida Boukhari, Magalie Demar

**Affiliations:** 1 Service de Dermatologie, Centre Hospitalier Andrée Rosemon, Cayenne, French Guiana;; 2 EA 3593, Ecosystèmes Amazoniens et Pathologies Tropicales, Université de Guyane, Cayenne, French Guiana;; 3 Centre Hospitalier Andrée Rosemon, Laboratoire Hospitalo-Universitaire de Parasitologie-Mycologie, Cayenne, French Guiana;; 4 Centres Délocalisés de Prévention et de Soins, Centre Hospitalier Andrée Rosemon, Cayenne, French Guiana;; 5 Laboratoire, Centre Hospitalier de l’Ouest Guyanais, Saint-Laurent-Du-Maroni, French Guiana

## Abstract

*Strongyloides stercoralis* and human T-lymphotropic virus 1 (HTLV-1) coinfections have been extensively reported in the literature, but the diagnosis and treatment of strongyloidiasis remains a challenge, particularly in HTLV-1 carriers. Our objectives were to evaluate the efficacy of a new PCR method for the detection of *S. stercoralis* in HTLV-1–positive patients. Stools were collected over a 1-year period across the endemic region of French Guiana, including remote forest areas. Two systems of real-time PCR were then used comparatively, with small subunit and specific repeat as respective targets, and compared with the results of microscopic examinations. One-hundred and twelve stool samples were included. Twenty-seven patients (24.1%) presented a positive HTLV-1 serology. The overall prevalence of strongyloidiasis among the 112 patients was 30% with small-subunit PCR and 11.6% with microscopic examinations. In the seropositive population, all tested stools were negative, whereas 51.2% were positive using small-subunit PCR. Thus, PCR allowed a much-improved sensitivity, particularly in HTLV-1 carriers. Among the two systems investigated, small subunit yielded better results than specific repeat PCR, with prevalence rates in HTLV-1 carriers of 51.2% and 22.2%, respectively. Therefore, PCR should be considered as a useful tool for the diagnosis of strongyloidiasis, particularly in HTLV-1 carriers who often present a light parasitic load due to erratic administration of anthelmintic drugs.

## INTRODUCTION

Human T-lymphotropic virus 1 (HTLV-1) infection and strongyloidiasis are two diseases that often share a common geographic distribution. French Guiana is known to harbor high levels of endemicity for both of them.^1^ Negative effects of coinfection have been extensively described in the literature.^2^ HTLV-1 infection increases the prevalence of strongyloidiasis,^3^ the rate of treatment failure,^3,4^ and the risk of hyperinfestation.^5^ On the other hand, several studies have highlighted the possible role of strongyloidiasis as a cofactor for the development of adult T-cell leukemia/lymphoma (ATLL).^6,7^

In 2000, Gabet et al.^8^ reported a higher proviral load in HTLV-1 carriers with *Strongyloides stercoralis* infection. This study included several patients from French Guiana, but involved only a small sample and did not compare *S. stercoralis* incidence between HTLV-1 seronegative and seropositive patients. Therefore, coinfection with HTLV-1 and *S. stercoralis* has not been specifically studied in French Guiana, although it has been evaluated in the French West Indies. In Martinique, 20% of individuals infected with *S. stercoralis* are coinfected with HTLV-1.^9^ In Guadeloupe, 31% of HTLV-1–positive subjects have *S. stercoralis* antibodies, as compared with 11% of negative donors.^10^ In French Guiana, the prevalence of strongyloidiasis can be as high as 16% in Amerindian communities.^1^ Concerning HTLV-1, a screening of blood donors in 2003 showed a seroprevalence of 1.3%^11^ in the overall population. This figure reached 8% in the Bushinengue (Maroon) community.^12^

As in many remote areas, prevalence of strongyloidiasis is possibly underestimated in French Guiana, as its diagnosis often relies on microscopic examinations, which are difficult to perform in isolated health centers. Indeed, techniques such as Baermann or agar plate culture are time-consuming and require several samples of fresh stools, which can be hard to collect in these remote communities.^13^ Therefore, there is a need for new techniques for the isolation of *S. stercoralis* in these settings. In 2009, results were published comparing two PCRs targeting the small-subunit (SSU) rRNA gene and *S. stercoralis*–specific repeat (RS) in fecal samples, with 100% specificity and a high sensitivity.^14^ Notably, a 2-fold increase in the detection was observed, when compared with the Baermann sedimentation method. However, this promising method has not been used to explore the issue of coinfection with HTLV-1.

Our objectives were to evaluate the utility of a PCR-based diagnosis of *S. stercoralis* in the remote areas of French Guiana, to compare the performances of two different probe systems (SSU and RS), and to evaluate the prevalence of *S. stercoralis* in the HTLV-1 seropositive population.

## METHODS

Stools were collected over a 1-year period at the hospitals of Cayenne and Saint-Laurent. Stools were included when positive for any helminthiasis, or when corresponding to patients with known HTLV-1 serological status, or when originating from any areas of French Guiana, including the health centers for remote areas. Three patients, who did not complain of any symptom and had never traveled to any endemic area, were used as negative controls.

Direct examination and Baermann test were performed for every patient. Results of this microscopic examination, eosinophil count, serological status for HTLV-1, age, gender, region of origin, and clinical symptoms were recorded. Stools were kept at −20°C until DNA extraction using Ultra Clean Fecal DNA kit^™^ (MO BIO^®^, Carlsbad, CA). Two systems of real-time PCR were then used comparatively, with SSU and RS as respective targets. Primers were synthesized using the sequences provided in the publication by Verweij et al.^14^ (GenBank accession numbers AY028262 and AF 279916). TaqMan exogenous internal positive control (Applied Biosystems^™^, Foster City, CA) was used to exclude the presence of PCR inhibitors. PCR was deemed negative when no amplification could be recorded or above a threshold of 40 cycle threshold (Ct).

## RESULTS

One hundred and twelve stool samples were included. Patients originated from the Upper Oyapock (46, 41%), the Maroni region (46, 41%), the Cayenne metropolitan area (17, 15.2%), and mainland France (3, 2.8%). Twenty-seven patients (24.1%) presented a positive HTLV-1 serology, all originating from the Maroni region. Among them, seven belonged to the Creole community, whereas 20 belonged to the Bushinengue community.

Results of microscopic examinations and PCR with both methods are presented in Table 1. In the HTLV-1–negative population, the estimated prevalence of strongyloidiasis with microscopic examination was significantly lower than that with SSU PCR (15.3% versus 21.2%). In the seropositive population, all tested stools were negative, whereas 51.2% were positive using SSU PCR. The overall prevalence of strongyloidiasis among the 112 patients was 30% with SSU PCR and 11.6% with microscopic examinations.

**Table 1:** Number of positive PCR with each target (SSU and RS) among the two populations (HTLV-1 positive and negative), compared with the results of microscopic examinations

		Positive PCR (*n*)			
	Microscopic examination	SSU PCR	Ct: Median (range)	RS PCR	Ct: Median (range)
HTLV-1 negative (*n* = 85)	Positive stools (*n* = 13)	13	28.3 (22–38.5)	12	34.5 (28.4–38.4)
	Negative stools (*n* = 72)*	5	36.5 (32.9–40)	0	
HTLV-1 positive (*n* = 27)	Positive stools (*n* = 0)	0	–	0	36.5 (33–40)
	Negative stools (*n* = 27)	14	33.3 (26.9–39.4)	6	

RS = specific repeat; SSU = small subunit.

* In 39 cases, microscopic examination was negative for *Strongyloides stercoralis* but positive for other helminthiasis; among these 39 cases, two had positive *S. stercoralis* PCR.

When comparing the two PCR targets, SSU was more sensitive than RS in both populations. Among the 27 patients with positive HTLV-1 serology and negative stools, SSU PCR allowed the detection of *S. stercoralis* in 14 of them, whereas only six were positive using the RS technique. In these patients, the mean Ct with SSU PCR and RS was, respectively, 33.33 (26.9–39.4) and 36.5 (33–40). Among the 72 patients with negative HTLV-1 serology and negative stools, SSU PCR allowed the detection of *S. stercoralis* in five of them, whereas RS was always negative.

## DISCUSSION

In this study, the prevalence of *S. stercoralis* determined by microscopic examinations (11.6%) was slightly higher than the prevalence rates previously reported in Amerindian communities in Brazil (5.6%)^15^ or Peru (8.7%).^16^ However, in a community-based study performed among the Wayampi Amerindians in French Guiana in 2002, *S. stercoralis* was detected in 16% of tested stools. In our study, it is noteworthy that the estimated prevalence was much higher when using PCR (30%) than with microscopic examinations (11.6%).

Indeed, the higher sensitivity of PCR allowed the detection of *S. stercoralis* in 17 stools with negative Baermann tests. This number was particularly significant in HTLV-1 seropositive patients (14 stools). This study confirms the high sensitivity of PCR for the detection of light infections that are missed by traditional microscopic examinations.^17^ A systematic review performed in 2012 found discordant results and suggested that PCR should be used only as a confirmation test.^18^ However, this study included comparisons with serology, whose specificity remains doubtful.^16^ Therefore, considering the results, PCR offers a much improved sensitivity, if the SSU system is used.

We compared SSU and RS techniques and our results were similar to those of Verweij et al., who reported a mean Ct of 28.1 with the SSU system in case of positive microscopic examination (28.3 in our study), with a much higher sensitivity than the RS system. In our study, all stools with positive SSU PCR presented lower Ct with the SSU than with the RS system. We report one case of positive microscopic examination and negative RS PCR, in a sample which contained only a few larvae. The SSU technique was positive in all cases of positive microscopic examinations. It also allowed the detection of *S. stercoralis* in five stools among the 72 HTLV-1–negative patients. All of these five patients had symptoms such as abdominal pain and diarrhea.

Concerning coinfection with HTLV-1 and strongyloidiasis, microscopic examination did not detect *S. stercoralis* in the stool of seropositive patients, when PCR was positive for 14 of them (51.2%). To the best of our knowledge, this study is the first one to compare the performances of PCR and microscopic examinations in this population. In a study in Martinique among patients with ATLL, 42% of stools were positive using the Baermann method, but only patients with abdominal pain or diarrhea were tested.^9^ In a screening performed in Belem, 14.3% of HTLV-1 patients were positive using microscopic methods, compared with 0% in our study.^19^ However, in this Brazilian study, all participants reported taking no recent anthelmintic treatment. Conversely, all our HTLV-1–positive patients presented negative microscopic examinations. A low level of parasitism is often observed in these patients who are frequently treated with anthelmintic drugs. PCR offers a better sensitivity and could be a useful tool in the follow-up of these patients. In our study, positive HTLV-1 patients all belonged to the Bushinengue or Creole communities, an expected result, as the other communities of French Guiana (White, Amerindians, etc.) are known to harbor very few virus carriers.^11,12,20^

Concerning the specificity of this PCR, Verweij et al. reported no false positives in their publication, using a large range of control DNA and stool samples. This high specificity was confirmed in our results (Table 1). Among HTLV-1 seronegative patients, 39 stools were positive for other helminthiasis, and only two of them (5.1%) had positive *S. stercoralis* PCR. There was no visible larva in the Baermann method for these two patients, who probably suffered from low-level parasitism. Even if these two results were to be deemed as false positive, specificity would still remain as high as 94.9%.

An argument often raised against PCR is its high cost and its availability, limited to large hospitals. In this study, as in previous works, the Baermann method was achievable on stool samples from some very isolated communities on the upper Maroni River.^8^ However, this collection implied many logistical hardships, as stool samples from these health centers for remote areas of French Guiana are always carried to the general hospital by boat, sometimes for several days, which hampers the conservation of live larvae. Because of logistical issues, stools are rarely collected three times, as recommended for the Baermann method. The high sensitivity of PCR allows easy detection with only one sample. The lack of trained personnel in Western French Guiana (Saint-Laurent) does not allow laboratories to perform microscopic examinations on a regular basis. Molecular biology, on the other hand, is routinely performed in Cayenne and Saint-Laurent. Therefore, this experimentation in French Guiana could be an example for other remote areas of endemic countries.

## CONCLUSION

Small-subunit PCR is a useful method for the diagnosis of *S. stercoralis* in HTLV-1 carriers. It greatly improves the detection rate, compared with microscopic examination. Its high sensitivity, even after the administration of anthelmintic drugs, allows a close follow-up of patients after treatment. It represents an efficient diagnostic tool for HTLV-1 carriers treated in a tropical, middle-income setting such as French Guiana. Coinfection with *S. stercoralis* and HTLV-1 could be even higher in seropositive patients than previously suggested, as the better sensitivity of PCR allowed us to detect *S. stercoralis* DNA in as much as 51.2% of seropositive patients.
